# Implementation of Glycan Remodeling to Plant-Made Therapeutic Antibodies

**DOI:** 10.3390/ijms19020421

**Published:** 2018-01-31

**Authors:** Lindsay D. Bennett, Qiang Yang, Brian R. Berquist, John P. Giddens, Zhongjie Ren, Vally Kommineni, Ryan P. Murray, Earl L. White, Barry R. Holtz, Lai-Xi Wang, Sylvain Marcel

**Affiliations:** 1 Metropolitan Nashville Police Department Crime Lab, 400 Myatt Drive, Madison, TN 37115, USA; Lindsay.bennett@nashville.gov; 2 Department of Chemistry and Biochemistry, University of Maryland, 8051 Regents Drive, College Park, MD 20742, USA; qyang1@umd.edu (Q.Y.); johnpgiddens@gmail.com (J.P.G.); 3 iBio CDMO, 8800 Health Science Center Parkway, Bryan, TX 77807, USA; bberquist@ibiocmo.com (B.R.B.); zren@ibiocmo.com (Z.R.); vkommineni@ibiocmo.com (V.K.); bholtz@ibioinc.com (B.R.H.); 4 Lonza Houston, Inc., 8066 El Rio St., Houston, TX 77054, USA; ryan.murray@lonza.com; 5 MDx BioAnalytical Laboratory, Inc., 5890 Imperial loop, Suite 12, College Station, TX 77845, USA; earl.white@mdxbiolabs.com

**Keywords:** glycan remodeling, therapeutic proteins, recombinant glycoproteins, *Nicotiana benthamiana*, *N*-glycosylation

## Abstract

*N*-glycosylation profoundly affects the biological stability and function of therapeutic proteins, which explains the recent interest in glycoengineering technologies as methods to develop biobetter therapeutics. In current manufacturing processes, *N*-glycosylation is host-specific and remains difficult to control in a production environment that changes with scale and production batches leading to glycosylation heterogeneity and inconsistency. On the other hand, in vitro chemoenzymatic glycan remodeling has been successful in producing homogeneous pre-defined protein glycoforms, but needs to be combined with a cost-effective and scalable production method. An efficient chemoenzymatic glycan remodeling technology using a plant expression system that combines in vivo deglycosylation with an in vitro chemoenzymatic glycosylation is described. Using the monoclonal antibody rituximab as a model therapeutic protein, a uniform Gal2GlcNAc2Man3GlcNAc2 (A2G2) glycoform without α-1,6-fucose, plant-specific α-1,3-fucose or β-1,2-xylose residues was produced. When compared with the innovator product Rituxan^®^, the plant-made remodeled afucosylated antibody showed similar binding affinity to the CD20 antigen but significantly enhanced cell cytotoxicity in vitro. Using a scalable plant expression system and reducing the in vitro deglycosylation burden creates the potential to eliminate glycan heterogeneity and provide affordable customization of therapeutics’ glycosylation for maximal and targeted biological activity. This feature can reduce cost and provide an affordable platform to manufacture biobetter antibodies.

## 1. Introduction

Therapeutic glycoproteins represent a predominant disease treatment category among biopharmaceuticals approved or in clinical development. N-linked glycosylation is a very important post-translational modification that can profoundly affect the stability and biological activities of proteins [1]. While the amino acid sequence of a recombinant protein is pre-defined, the structure of the N-linked oligosaccharide decorating proteins varies depending on host species, host cell type, and environmental growth conditions [2]. All non-engineered eukaryotic cells share a common *N*-glycosylation core, while terminal and lateral oligosaccharide residues are specific to each expression system. The processing of protein *N*-glycosylation within the cell endomembrane system is complex and often results in the production of a heterogeneous population of glycoforms [3]. Control over the complexity and variability of protein *N*-glycosylation, although desired in therapeutic protein manufacturing, remains a challenge. Design of a manufacturing process allowing for precise regulation of protein *N*-glycosylation is highly advantageous and urgently needed, as *N*-glycosylation often profoundly affects biological efficacy and pharmacokinetic properties of the manufactured drug substance [4,5,6,7]. Indeed, numerous preclinical studies have supported the strong relationship between the glycosylation structure decorating therapeutic proteins and their clinical properties, including protein stability, solubility, circulatory half-life, and efficacy [8,9,10,11,12,13,14,15,16]. A well-characterized example of an *N*-glycosylation pattern affecting therapeutic efficacy was shown with the removal of the α-1,6-fucose residue attached to the innermost *N*-acetylglucosamine (GlcNAc) of the *N*-glycosylation core present on some anticancer antibodies such as rituximab and trastuzumab. Afucosylated or low-fucose content antibodies exhibited enhanced antibody-dependent-cell-cytotoxicity (ADCC) in vivo when compared to their fucosylated counterparts [9,14,16,17,18,19]. As further proof of the importance of *N*-glycosylation pattern affecting therapeutic activity, the first glycoengineered products, afucosylated anti-CCR4 mogamulizumab (POTELIGIO^®^, Kyowa Hakko Kirin) and anti-CD20 obinutuzumab (Gazyva^®^, Roche) were approved for human use in Japan and in the United-States in 2012 and 2013 respectively.

Several manufacturing approaches have been considered to modify the host *N*-glycosylation machinery, with the goal of exploiting the benefit of specific glycosylation profiles and generating therapeutics with targeted biological functions. These included modulation of cell growth conditions [2], silencing or overexpression of glycan processing enzymes [20,21,22], complete or partial removal of glycans [23,24,25], and in vitro glycosylation remodeling [26,27]. While glycan engineering technologies using a α-1,6-fucosyltransferase (FUT8) knockout Chinese hamster ovary (CHO) cell line [28] or plant α-1,3-fucosyltransferase/β-1,2-xylosyltransferase (∆FT/XT) knockdown lines [29,30,31,32] significantly reduced glycosylation heterogeneity, there remained a significant degree of non-uniformity in the final product. Likewise, modulation of cell growth conditions remains prone to batch-to-batch product variability [33,34]. In addition, knock-in strategies have been employed to introduce or modify the sialylation pathway and provide recombinant proteins with human-type sialylation; offering higher circulatory half-life and reduced product immunogenicity [35,36,37]. This type of approach generally enhanced human-type protein sialylation, but also increased glycoform heterogeneity.

Consequently, in vitro glycan remodeling was developed to offer the advantage of adding customized oligosaccharides on recombinant proteins to produce a single uniform glycoform [26,38,39]. In particular, using the endoglycosidase-catalyzed glycan remodeling approach, N-linked glycoproteins are enzymatically deglycosylated such that the innermost GlcNAc or Fucα-1,6GlcNAc remains on the asparagine residue of the protein [40,41,42,43]. This approach allows for an *N*-glycan oxazoline substrate to be transferred en bloc onto that remaining GlcNAc of recombinant proteins to reconstitute homogeneous glycoproteins. In vitro glycan remodeling technology offers the benefit of producing uniform and customized glycoproteins. Recently the glycan remodeling approach has been applied to produce homogeneous antibody glycoforms for structural and functional studies [44,45,46,47,48,49]. Although the concept of glycan remodeling has been demonstrated in vitro, it has not yet been applied to any recombinant protein manufacturing platform.

A simplified glycan remodeling strategy is presented, where the initial in vitro deglycosylation step has been incorporated into the host expression system, thereby opening potential to the affordable manufacturing of glycoengineered therapeutic proteins. Rituximab was chosen to remodel, as a model monoclonal antibody, and an afucosylated antibody glycoform with two terminal galactose residues (A2G2 glycoform, oxford glycan nomenclature [50]) was generated. The resulting antibody displayed enhanced ADCC activity using a transient expression protocol developed for large-scale therapeutics manufacturing in the well-characterized *Nicotiana benthamiana* plant system. Incorporating protein glycan moiety remodeling into this plant expression system demonstrates the production of a single glycoform rituximab harboring human-like *N*-glycosylation and no plant-specific sugar residues. This novel approach simplifies an expensive glycan remodeling procedure facilitating a cost-effective and scalable process.

## 2. Results

### 2.1. Rituximab Is Deglycosylated In Vivo

**Figure 1:**
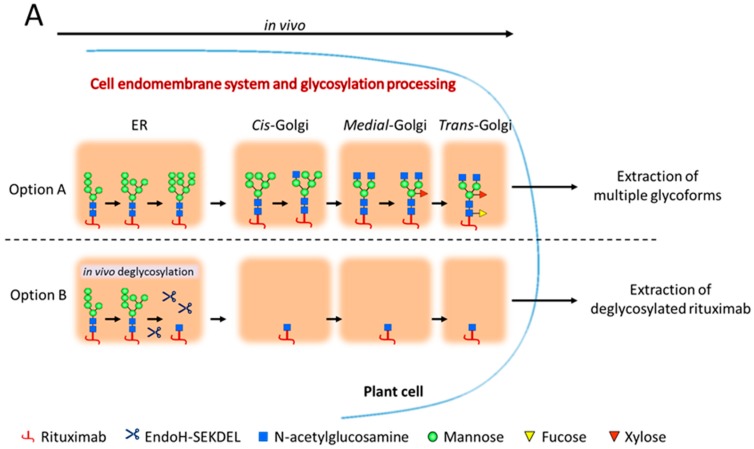

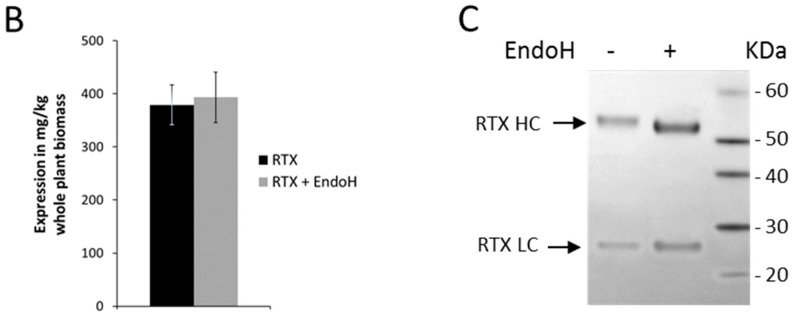
(**A**) Schematic representation of in vivo *N*-glycan processing of recombinant proteins in standard plant expression systems (Option A) and when co-expressed with EndoH (Option B). The Option B pathway illustrates the in vivo deglycosylation strategy employed for rituximab custom glycosylation where high-mannose glycans attached to the protein of interest are cleaved off by EndoH, leaving a deglycosylated substrate for in vitro transglycosylation; (**B**) plots of rituximab expression levels in mg/kg of plant biomass. Protein was harvested from plants expressing rituximab alone (black bar) or coexpressing rituximab with EndoH (grey bar) (Values are means ± SEM, *n* = 4); (**C**) SDS-PAGE showing rituximab under reduced conditions, with or without EndoH coexpression. The light chain (RTX LC) species for both samples migrated similarly, while the heavy chain (RTX HC) from plants coexpressing EndoH underwent an increased mobility relative to the rituximab alone, indicating a decrease in molecular weight.

Rituximab was transiently expressed in *Nicotiana benthamiana* plants using vacuum infiltration. Proteins were fused to a signal peptide to allow for post-translational modification (i.e., *N*-glycosylation) in the plant endomembrane system and secretion to the apoplast (Option A, Figure 1A). To produce deglycosylated antibody retaining a single *N*-acetylglucosamine (GlcNAc), rituximab was co-expressed in plants with the endoglycosidase H (EndoH). EndoH was targeted to the plant cell endoplasmic reticulum (ER) using the ER-retrieval sequence SEKDEL to ensure that its activity remains in the ER where high-mannose glycans (the EndoH target) are processed (Option B, Figure 1A). Rituximab, when expressed alone (_Nb_RTX), accumulated at 379 mg/kg of plant biomass. When co-expressed with EndoH (_Nb_RTX^GlcNAc^), the antibody accumulated at 394 mg/kg of plant biomass (Figure 1B), which indicated that the accumulation of rituximab was not impeded by the co-expression with the endoglycosidase (*p* = 0.99). Rituximab was purified from plant host cell proteins by protein A affinity chromatography. The efficiency of in vivo deglycosylation was first assessed at this stage by SDS-PAGE in reducing conditions (Figure 1C). Rituximab heavy chain co-expressed with EndoH (lane EndoH+, Figure 1C) migrated at a lower molecular weight than rituximab heavy chain expressed alone (lane EndoH−, Figure 1C), suggesting efficient removal of core oligosaccharides from the antibody. Subsequent cation exchange chromatography was then employed to separate _Nb_RTX^GlcNAc^ from remaining non-hydrolyzed rituximab forms, yielding fractions exceedingly enriched for _Nb_RTX^GlcNAc^.

**Figure 2:**
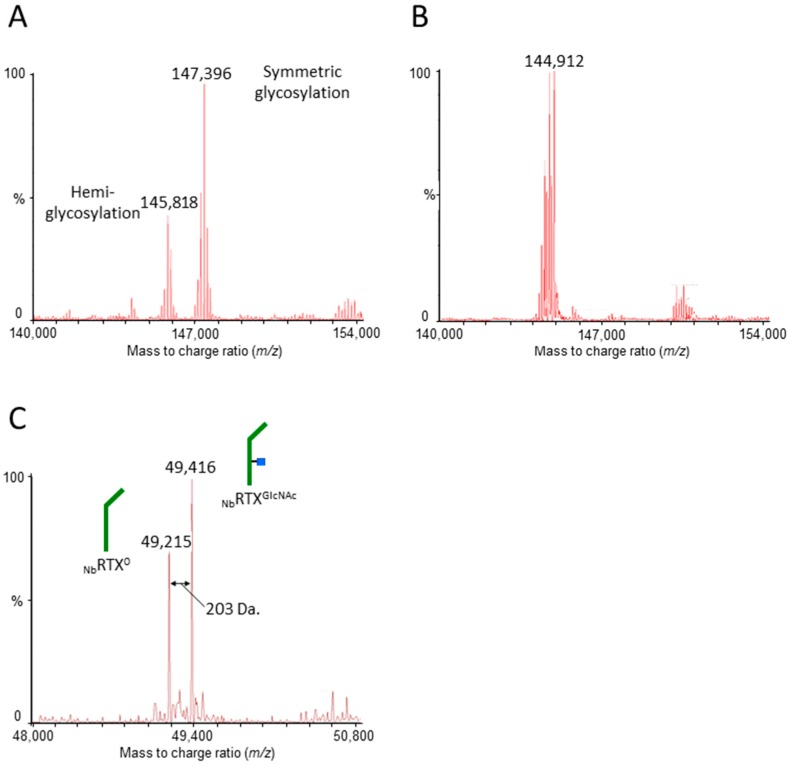
Nano liquid chromatography-electrospray ionization-quadrupole time of flight-mass spectrometry (NanoLC-QTOF-MS) analysis of plant-made rituximab. (**A**) Deconvoluted electrospray ionization (ESI)-mass spectrum of purified _Nb_RTX analyzed under non-reducing conditions showing fully glycosylated and hemiglycosylated rituximab; (**B**) Deconvoluted ESI-mass spectrum of purified _Nb_RTX^GlcNAc^ analyzed under non-reducing conditions showing a decrease in molecular weight; (**C**) Deconvoluted ESI-mass spectrum of in vivo deglycosylated plant-made rituximab analyzed under reducing conditions. Note the mass shift between non-glycosylated rituximab heavy chain _Nb_RTX^0^ (calculated *m*/*z* 49,217) and deglycosylated rituximab heavy chain _Nb_RTX^GlcNAc^ (calculated *m*/*z* 49,420). As comparison, the NanoLC-QTOF-MS analysis of Rituxan^®^ is provided in Figure S2.

The profiles of _Nb_RTX and _Nb_RTX^GlcNAc^ glycoforms were further characterized by NanoLC-QTOF mass spectrometry (MS) to confirm the mass-shift observed by SDS-PAGE. The MS analysis of purified _Nb_RTX revealed the presence of hemiglycosylated rituximab species (at about 145,818 Da) besides fully glycosylated rituximab (at about 147,396 Da; Figure 2A). The same analysis performed on rituximab co-expressed with Endo-H (_Nb_RTX^GlcNAc^) revealed a shift of molecular weight to about 144,912 Da (Figure 2B) corresponding to the calculated mass of the non-glycosylated antibody plus the mass of two *N*-acetylglucosamine residues (calculated mass of 144,910.4 Da; Tables S1–S3 and Figure S1). Note that the calculated mass of plant-made rituximab differs slightly from Rituxan^®^. Indeed, plant-made rituximab also exhibits *N*-terminal pyroglutamate residues (Figure S1) as for Rituxan^®^, but does contain the C-terminal lysine contrary to Rituxan^®^ (Table S1). Also, Rituxan^®^ holds an alanine residue at position 219 while the plant-made rituximab has a valine residue (as in the original human IgG1). However, no other modifications, such as hydroxyproline or methionine oxidation, were observed. In reducing conditions, the MS analysis of _Nb_RTX^GlcNAc^ depicted the isolation of non-glycosylated heavy chain (_Nb_RTX^0^ heavy chain; 49,215 Da; Figure 2C) and deglycosylated heavy chain (_Nb_RTX^GlcNAc^ heavy chain; 49,416 Da; Figure 2C). The presence of _Nb_RTX^0^ heavy chain offered a direct comparison with _Nb_RTX^GlcNAc^ heavy chain as the mass of _Nb_RTX^GlcNAc^ heavy chain was approximately 203 Da (molecular weight of one GlcNAc residue) greater than that of _Nb_RTX^0^ heavy chain, confirming the complete removal of N-linked glycans at the predicted Endo-H cleavage site (Figure 2C) and the absence of α-1,3 fucose residue. It is recognized that non-glycosylated heavy chain was originated from hemiglycosylated rituximab and appeared to exhibit similar chromatographic properties as deglycosylated rituximab. Rituximab heavy chain species with intact *N*-glycan structures were not detected, indicating efficient chromatographic separation of _Nb_RTX^GlcNAc^ from non-hydrolyzed rituximab forms. The extraction and purification of a deglycosylated product lacking the fucose residue attached to the innermost GlcNAc was accomplished by designing the in vivo deglycosylation step to occur early during the plant glycosylation maturation.

### 2.2. Complete Chemoenzymatic Transfer of Complex-Type Glycan Oxazoline onto Plant-Made Rituximab

**Figure 3:**
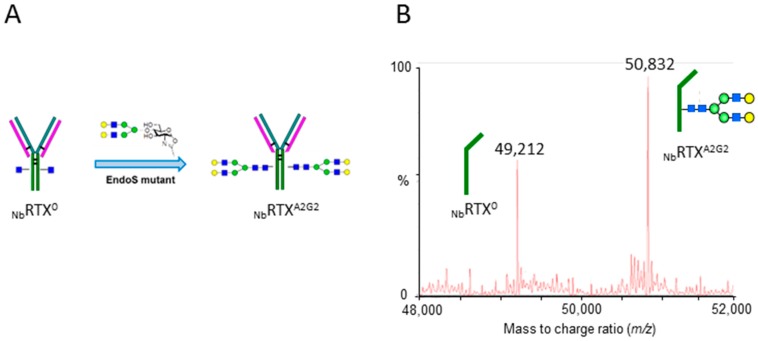
Chemoenzymatic transglycosylation of plant-made rituximab. (**A**) Schematic representation of the chemoenzymatic transglycosylation reaction; (**B**) NanoLC-QTOF-MS analysis of reglycosylated plant-made rituximab. Deconvoluted ESI-mass spectrum of reglycosylated plant-made rituximab with the A2G2 glycan (_Nb_RTX^A2G2^ heavy chain, calculated *m*/*z* 50,840) analyzed under reducing conditions.

An endoglycosidase S mutant (EndoS D233Q) has been previously selected for the specific activity of rapid and robust transglycosylation of oxazoline glycans onto a single core GlcNAc attached to the protein asparagine residue [40]. Oxazoline glycans were prepared from pure sialylglycopeptide (SGP) isolated from chicken egg yolk, following the previously reported method [51,52]. In addition, a scalable acetone-water solution-based extraction has also been published capable of producing gram quantities of oxazoline glycan reagents [53]. Plant-derived _Nb_RTX^GlcNAc^ was used as the acceptor in the EndoS D233Q mediated transglycosylation reaction which enables the transfer of a complex *N*-glycan to the exposed GlcNAc residue to produce a A2G2 glycoform (Figure 3A). Liquid chromatography-electrospray ionization-tandem mass spectrometry (LC-ESI-MS) analysis confirmed the completion of the reaction showing the formation of a rituximab glycoform of 50,832 Da which corresponds to the A2G2 glycoform of the heavy chain of the IgG. Mass spectrometry analysis also revealed the specificity of the transglycosylation for the _Nb_RTX^GlcNAc^ species, as the non-glycosylated heavy chain fraction, which does not carry GlcNAc acceptor at position Asn^297^, remained unprocessed (Figure 3B).

### 2.3. Cell Surface CD20 Binding of _Nb_RTX^A2G2^

**Figure 4:**
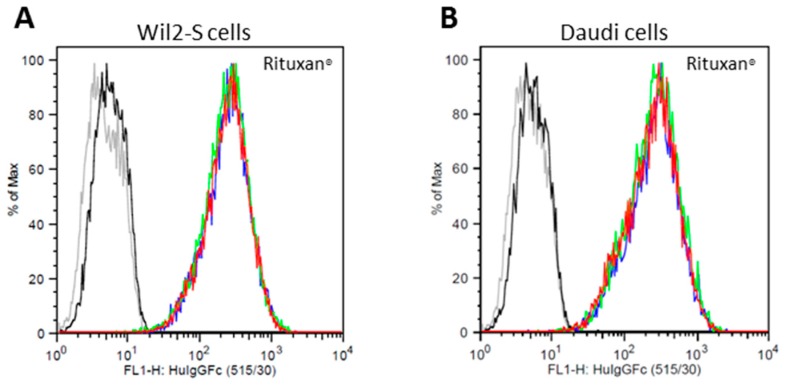

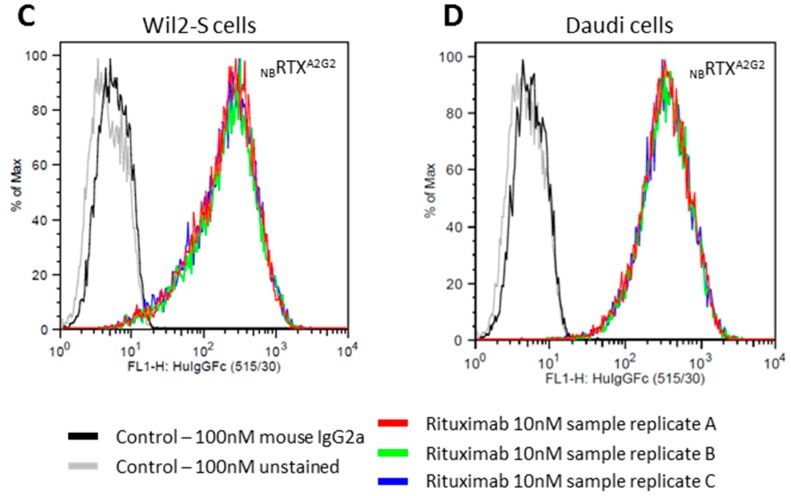
Binding of Rituxan^®^ (**A**,**B**) and _Nb_RTX^A2G2^ (**C**,**D**) to Wil2-S and Daudi cells analyzed by flow cytometry. Rituxan^®^ and _Nb_RTX^A2G2^ were used at a concentration of 10 nM. All rituximab samples were measured in triplicates (red, green and blue lines). FITC-labeled mouse IgG2a (black line) and unstained cells (grey line) were used as controls. The X-axis represents the fluorescent signals of FITC whereas the Y-axis presents % of cell count.

Rituximab mechanism of action relies on binding to the lymphoma cell marker CD20 before triggering cell cytotoxicity. Prior to performing CD20 binding assays, the percentage of CD20 antigen on cell surface of Wil2-S and Daudi lymphoma cells, standard lymphoma cell lines used for CD20 binding assays in oncology research, was first quantitated by flow cytometry using FITC-labeled mouse anti-human CD20 antibodies. It was found that 98.7% of Wil2-S and 99.4% of Daudi cells were CD20 positive under culture conditions (Figure S3). Since cells retained high expression of CD20, they were used for binding and cytotoxicity assays. The binding assay was carried out with 10 nM of _Nb_RTX^A2G2^ and Rituxan^®^ using flow cytometry. _Nb_RTX^A2G2^ revealed similar binding affinity to Rituxan^®^ when both Wil2-S and Daudi cells were used (Figure 4). Similar binding profiles were observed when 50 nM and 100 nM of rituximab samples were used. This demonstrated that the A2G2 glycoform status and the reglycosylation procedure did not impede the antibody binding capacity to target cells.

### 2.4. Enhanced ADCC Response by _Nb_RTX^A2G2^

**Figure 5:**
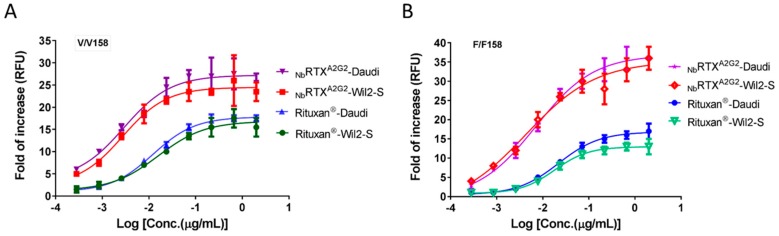
Antibody-dependent-cell-mediated cytotoxicity (ADCC) activity of Rituxan^®^ and _Nb_RTX^A2G2^ with (**A**) V/V 158 FcγRIIIa (high affinity) and (**B**) F/F 158 FcγRIIIa (low affinity) variant effector cells (engineered Jurkat cells with FcγRIIIa receptor). All experiments were carried out using human B lymphoma WIL2-S cells and Daudi cells as target cells. The effector cell: target cell ratio was 10:1. Data are expressed as fold of ADCC increase. Values represent mean ± S.D. for triplicate analyses.

**Figure 6:**
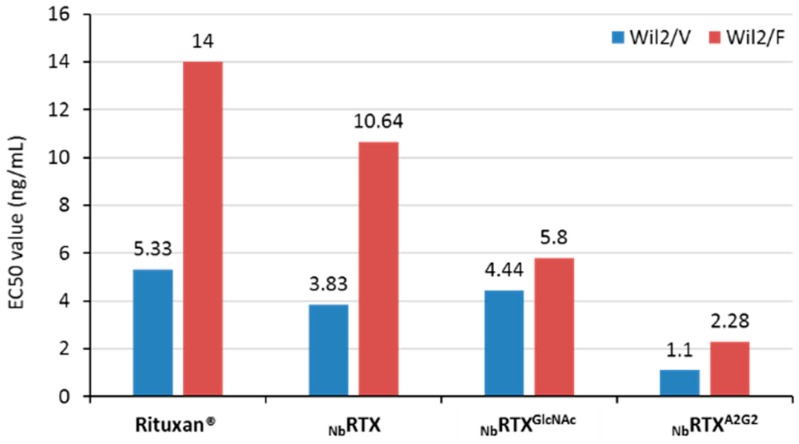
Comparison of EC50 values of Rituxan^®^, plant-made rituximab (_Nb_RTX), deglycosylated plant-made rituximab (_Nb_RTX^GlcNAc^), and reglycosylated plant-made rituximab (_Nb_RTX^A2G2^) determined by the ADCC Reporter Bioassay using Wil2 V/V158 (Wil2/V) and F/F158 (Wil2/F) variant cells. The calculated EC50 values are shown above the respective histogram bars.

Antibody-dependent cell-mediated cytotoxicity (ADCC) is an important mechanism of action of anti-cancer antibodies. ADCC assays are commonly performed using peripheral blood mononuclear cells (PBMCs), natural killer (NK) cells or engineered cell lines as effector cells. Analysis of ADCC efficacy of plant-made _Nb_RTX^A2G2^ was carried out using the ADCC reporter bioassay with Wil2-S and Daudi target cells along with engineered Jurkat cells expressing FcγRIIIa receptor. Engineered effector cell lines provide more consistent results in ADCC assays with less variability compared to primary effector cells and PBMC [54]. The impact of effector cells expressing different CD16a allotypes (FcγRIIIa receptor) on the ADCC activity of _Nb_RTX^A2G2^ and Rituxan^®^ was evaluated. The presence of a valine (V) versus a phenylaniline (F) at position 158 of FcγRIIIa/CD16a improves the affinity for IgG and is associated with higher ADCC efficacy. Both high affinity V/V 158 variant and low affinity F/F 158 variant effector cells were used. As shown in Figure 5, EC_50_ values of plant-made _Nb_RTX^A2G2^ were substantially higher than Rituxan^®^, regardless of the phenotype of the effector cells (V/V 158 or F/F 158 variant) used. Dose response curves of _Nb_RTX^A2G2^ exhibited a leftward shift and significantly higher upper asymptote from those of Rituxan^®^, which indicated improved cytotoxic activity of _Nb_RTX^A2G2^ over Rituxan^®^. When V/V 158 variant effector cells were used, _Nb_RTX^A2G2^ triggered a 5.8-fold increase in ADCC response when using Wil2-S cells (EC_50_ 2.7 ng/mL) and a 4-fold increase using Daudi cells (EC_50_ 2.9 ng/mL) over Rituxan^®^ (EC_50_ 15.7 ng/mL and 12 ng/mL respectively). Similarly, when F/F 158 variant effector cells were used, the ADCC response triggered by _Nb_RTX^A2G2^ was stronger than Rituxan^®^ whether Wil2-S cells (EC_50_ of 4.4 ng/mL and 18 ng/mL respectively) or Daudi cells (EC_50_ 7 ng/mL and 22 ng/mL respectively) were used (Figure 5). A second ADCC assay was performed using Wil2-S cells (F/F 158 and V/V 158 variants) where _Nb_RTX^A2G2^ and Rituxan^®^ were compared to _Nb_RTX and _Nb_RTX^GlcNAc^. The resulting EC50 values are summarized in Figure 6. The superior ADCC potency of _Nb_RTX^A2G2^ over Rituxan^®^ or the non-engineered plant-made rituximab (_Nb_RTX) was confirmed.

## 3. Discussion

The cost of monoclonal antibody production remains high despite higher yields in CHO-based manufacture. With antibodies targeted to smaller patient populations and smaller batch sizes, the complexity of facilities, even with single-use technology does not offer substantive economies of scale. CHO glyco-engineering is even more difficult. In some cases, the capital and cost of goods constraints prevent the establishment of antibody manufacturing facilities in developing countries where it is badly needed. The plant transient expression platform using *N. benthamiana* was chosen as it offers a potential cost-effective alternative to other recombinant protein manufacturing techniques [55,56] to counterbalance the downstream process cost associated with the transglycosylation technology. Successful antibody transglycosylation was described earlier using purified antibodies derived from mammalian cell culture [40,41,57]. However, introducing glycan remodeling into a scalable manufacturing process remained challenging because of the additional cost associated with in vitro de- and re-glycosylation, and the introduction of in-process enzymes. An alternative to the in vitro deglycosylation step of the glycan remodeling technology was designed. A combined glycan remodeling process was developed that produced deglycosylated antibody acceptors in vivo, followed by in vitro chemoenzymatic transglycosylation using oxazoline glycans as donors to generate single antibody glycoforms harboring the human afucosylated A2G2 glycan. Furthermore, introducing the deglycosylation step in plant reduces process steps and time, which will significantly reduce cost at scale. To further control cost, oxazoline glycans were chosen as they are derived from raw material available in large quantities (chicken egg yolk powder) and can be prepared at a rather low cost. The large-scale preparation of the sialoglycopeptide (SGP) and the glycan oxazoline derivative was exemplified by two recent reports [53,58].

Most therapeutic products on the market are well characterized at the amino acid level, but still consist of a pool of several glycoforms and therefore their manufacturing remains under constant regulatory pressure to provide a truly well-characterized protein. The nature of oligosaccharides decorating therapeutic proteins is critical for determining pharmacokinetic properties, as previously described for afucosylated anti-cancer antibodies [9,14,16,17,18]. In fact, when the *N*-glycosylation and the clinical performance of the corresponding therapeutic protein are linked, the *N*-glycosylation structure is considered as a Critical Quality Attribute (CQA) [6] and must be controlled and characterized during the therapeutic manufacturing process to assure product consistency for maximum efficacy and safety. Developing a scalable and more affordable method to produce therapeutics with defined preselected *N*-glycosylation will address important therapeutic, manufacturing and regulatory considerations including: (i) reduction of immunogenic reactions caused by non-human sugar residues; (ii) targeting of specific protein activities through customized *N*-glycosylation; (iii) control and maintenance of therapeutic quality attributes during manufacturing processes; (iv) development of biosimilar and biobetter products; and (v) easier regulatory approval process.

A co-expression strategy was implemented where antibodies were expressed with the enzyme endoglycosidase-H (EndoH) from *Streptomyces plicatus.* EndoH was targeted to the endoplasmic reticulum (ER) by fusing the ER retrieval signal (SEKDEL) to the C-terminus of the enzyme. Thus, ER-retained EndoH would hydrolyze high-mannose glycans from newly formed glycoprotein as they mature along the plant endomembrane system and before the addition of the plant-specific α-1,3 fucose residue to the protein glycosylation core. Rituximab expressed either alone or with EndoH accumulated at similar levels in the plant and high levels (>300 mg/kg plant biomass) of recombinant antibodies, purified as deglycosylated substrates containing a single GlcNAc competent for in vitro transglycosylation, were obtained. Deglycosylated antibodies were recovered by protein A chromatography followed by cation exchange chromatography to separate further the deglycosylated antibody from non-hydrolyzed forms. Although this process was designed for scale-up, further optimization of the in planta deglycosylation would allow for an increase in deglycosylated antibody recovery and simpler downstream process. Similarly, a certain degree of hemiglycosylation was observed on rituximab produced in plants, which at the current stage of the method development need further characterization. For comparison, Rituxan^®^ only exhibit fully glycosylated species (Figure S2) as expected for an optimized and approved product [48,59,60,61]. The biology affecting the degree of antibody hemiglycosylation in plants has not been studied and remains unknown. However, it is likely that plant growth conditions, light chain/heavy chain expression kinetics and other environmental conditions influence this phenomenon as well as glycosylation site occupancy and in planta deglycosylation efficiency. To that end, expression optimization has been initiated to improve deglycosylation of the target protein in planta and initiate a scale-up strategy for the method. Data resulting from upstream and downstream process optimization will help unravel the complexity behind glycosylation of recombinant protein produced in plants and will be reported when a systematic analysis will be completed. A recent study showed that the human granulocyte-macrophage colony-stimulating factor (GM-CSF) and the GA101 antibody processed in vivo by the GlycoDelete technology were efficiently secreted [25], supporting the assumption that _Nb_RTX^GlcNAc^ would be secreted as well. The final glycan remodeled rituximab (_Nb_RTX^A2G2^) bound to CD20 as well as the commercial Rituxan^®^ and elicited a much stronger ADCC response in standard therapeutic activity/efficacy assays; criteria for establishing _Nb_RTX^A2G2^ as a *bona fide* biobetter. This is consistent with other reports demonstrating ADCC enhancement by modulating the glycosylation profile of the monoclonal antibody [9,16,17,62,63,64]. It also supports the fact that in planta deglycosylation is not likely to affect antibody folding as the final reglycosylated product demonstrated similar or better binding to CD20 and FcγRIIIa receptor as the innovator protein.

Moving beyond afucosylated monoclonal antibodies, other therapeutics may benefit from customizing their glycosylation status to include recombinant lysosomal enzyme for better cellular uptake during enzyme replacement therapies [65,66,67], anti-viral neutralizing antibody [12,68], and sialylated recombinant proteins (with terminal *N*-acetylneuraminic acid residues) to provide additional anti-inflammatory activity [40,69], or increased protein half-life [70,71].

## 4. Materials and Methods

### 4.1. Construction of Expression Vectors

The genetic sequences of antibodies’ heavy and light chains and Endoglycosidase H (GenBank: AAA26738.1) were each fused to the barley α-amylase signal peptide sequence (GenBank: CAX51374.1). For rituximab sequences, the DrugBank accession DB00073 was used where a valine residue at position 219 (original to human IgG1) was described while in the original patent (US patent No. 5736137), Rituxan^®^ has an alanine residue substitution at position 219. Antibodies’ heavy and light chain genes were cloned into magnICON^®^ TMV- and PVX plant expression vectors (Icon Genetics GmbH, Halle, Germany) [72], respectively. The *Endoglycosidase H* gene was fused to genetic sequences of the endoplasmic reticulum (ER) retrieval peptide SEKDEL and the epitope Flag tag at the 3’ end before being cloned into the binary vector pGREENII (John Innes Center, Norwich, UK; GenBank: EF590266.1) [73]. All DNA sequences were codon optimized for expression in *Nicotiana benthamiana* and synthesized by ATUM (Newark, CA, USA) for the antibody genes and Eurofins MWG operon (Louisville, KY, USA) for the *Endoglycosidase H* gene. All expression vectors were then mobilized separately into *Agrobacterium tumefaciens* strain GV3101 by electroporation. The vector pGREENII containing the *Endo-H* gene was mobilized in *Agrobacteria* together with the helper vector pSOUP (John Innes Center, Norwich, UK) [73].

### 4.2. Protein Expression

To express deglycosylated antibody acceptors in planta, the antibody’s light and heavy chains were co-expressed with the EndoH enzyme as follow. Individual *Agrobacetrium* cultures carrying plant expression vectors were grown in LB media (AMRESCO, Solon, OH, USA) at 28 °C, 225 rpm for 2 days. Agrobacterium cultures were then mixed together in 5 mM MES buffer, pH 5.5 and diluted to a final OD_600nm_ of 0.01 for the antibodies’ light and heavy chain *Agrobacterium* clones and to a final OD_600nm_ of 0.07 for the *Endo-H Agrobacterium* clone. Five-week-old plants (grown in growth chambers at ~26 °C with an 18 h light/6 h dark photoperiod under LED lighting (Illumitex, Austin, TX, USA)) were then immersed into the solution of diluted *Agrobacteria* and infiltrated by applying a vacuum of 23 inHg for 3 min. Agro-infiltrated plants were incubated under constant LED light at ~22 °C with relative humidity of ~50% for recombinant protein production. Antibody accumulation was measured in crude extract and in process samples by BioLayer Interferometry (BLI) using protein A sensors on a BLItz instrument (Pall ForteBio, Fremont, CA, USA).

### 4.3. Protein Purification

After 6 days of post infiltration, plants (leaves and stems) were harvested and total soluble proteins were extracted in 2 volumes (*w*:*v*) of extraction buffer (50 mM sodium phosphate, 60 mM ascorbic acid, 5 mM EDTA, 1mM PMSF, pH 8.0), centrifuged at 14,000× *g* for 20 min at 4 °C, and clarified by 1.2/0.2 µm filtration. Clarified extracts were loaded onto MabSelect SuRe resin (GE Healthcare Life Sciences, Piscataway, NJ, USA) to collect total plant-produced antibodies. Antibodies were washed with 10 CV of 25 mM Tris, 0.2 M NaCl, 1 mM EDTA, 0.01% PS80; pH 7.5 and eluted with 4 CV of 100 mM NaOAc, 50 mM NaCl; pH 3.3. Elution fractions were pooled and then subjected to cation exchange chromatography using Fractogel EMD SO_3_^−^ resin. Antibodies were washed with 30 mM NaOAc, pH 6.0 and eluted with a linear salt (0 to 1 M NaCl) gradient over 20 CV. Pure antibody samples were analyzed by SDS-PAGE using a 4–10% Bis-Tris gradient NuPAGE^®^ gel (Life Technologies, Carlsbad, CA, USA) under reducing conditions. 

### 4.4. NanoLC-QTOF Mass Spectrometry

Samples were dialyzed against purified water for 1 h at room temperature using a 10,000 Da MWCO membrane. Following dialysis, samples were diluted to a concentration of 0.5 mg/mL with 0.1% formic acid in water. For reduced analysis, dithiothreitol was added to the samples to a final concentration of 25 mM and incubated for 30 min at 37 °C. Samples were allowed to cool to room temperature before injecting 2–5 µL on to a 100 mm × 0.3 mm, 3.5 µm C8 column (Waters, Milford, MA, USA). Reversed phase chromatography was performed with a gradient of 95% water: 5% acetonitrile: 0.1% formic acid as mobile phase A and 95% acetonitrile: 5% water: 0.1% formic acid as mobile phase B. The column was held at 10% B for 5 min, then programmed to 90% B over 20 min and held at 90% B for 2 min with a constant flow rate of 10 µL/min on a Waters CapLC HPLC. Pre-column splitting was employed to achieve acceptable nanoLC flowrates (~600 nL/min) for interface with a nano-electrospray ionization (ESI) source. Mass spectrometry was performed on a Micromass Q-TOF Micro (quadrupole time-of-flight mass spectrometer) operating in the full scan positive-ion mode. Continuum mass spectra were acquired over the mass range of 500–4000 amu with a scan time of 1.8 s and an inter-scan delay of 1 s. The capillary, sample cone and extraction cone voltage were 3654; 30 and 2.5 V, respectively. The source temperature was held at 120 °C, the cone N_2_ gas flow rate was 50 L/h and the collision energy was held constant at 10 V with a collision gas pressure of 10.2 PSI. The LC-MS analysis of transglycosylation product was performed on an LXQ Linear Ion Trap (Thermo Scientific, Waltham, MA, USA) with an Agilent Poroshell 300SB-C8 column (5 µm, 75 × 1 mm). The IgG antibody samples were treated with 50 mM TCEP (tris(2-carboxyethyl)phosphine) at room temperature for 20 min then subjected to LC-MS analysis. The analysis was performed at 60 °C eluting with a linear gradient of 25–35% MeCN containing 0.1% formic acid within 10 min at a flow rate of 0.40 mL/min. The LC-MS analysis of IdeS protease [74] treated antibody glycoforms was performed in the same manner but included a 1-hr incubation with IdeS at 37 °C prior to TCEP (tris(2-carboxyethyl)phosphine) treatment.

### 4.5. Peptide Mapping/Post-Translational Modification Analysis by LC-MS

The purified Rituximab samples were adjusted to 0.1 mg/mL by 50 mM Tris, pH = 8.0 and denatured by 50 mM ammonium bicarbonate containing 8 M Urea, pH = 8. The denatured samples were reduced by adding freshly prepared dithiothreitol (DTT) to a final concentration of 20 mM for reduction and incubated at 60 °C for 1 h. The reduced samples were alkylated by adding freshly prepared Iodoacetamide to a final concentration of 40 mM and incubated for 30 mins in dark at room temperature. The alkylation reaction was then quenched by adding DTT to 20 mM. The alkylated samples were then diluted 8 times with 50 mM ammonium bicarbonate. The diluted samples were digested by adding 5 µg trypsin protease followed by overnight incubation at 37 °C. The digestion reaction solution was exchanged with 50 mM ammonium bicarbonate buffer. The subsequent analysis by mass spectrometry was performed using a SCIEX tripleTOF 5600 instrument within the same day of sample preparation. The peptic digests of rituximab samples were applied to a 2.1 mm × 50 Synergi 4 µm fusion RF 80 Å LC Column (Phenomenex Corporate, Torrence, CA, USA) using 0.1% (*v*/*v*) formic acid in water at a flow rate of 200 μL/min. The peptides were eluted using a gradient of 2–85% acetonitrile over the course of 30 min. A blank solution using acetonitrile was utilized between each sample to avoid peak carry-over between runs. The mass spectrometer was calibrated using PPG positive ion calibration solution from SCIEX (SCIEX, Framingham, MA, USA). The eluted peptides were introduced into the mass spectrometer and analyzed using the mass spectrometer in positive ion mode with the following settings: High sensitivity mode, scan range 300–3000 *m*/*z*, Accumulation Time 250 ms, GS1 = 45, GS2 = 50, CUR = 30, source temperature = 500 °C and ISVF = 4800. Charge states from 2 to 5 were accepted, and targeting ions were excluded for 3 s. MS/MS data were obtained using the CID with rolling collision energy. The peptides were identified using the software PEAKS studio (Bioinformatics Solutions Inc., Waterloo, ON, Canada) with a mass tolerance of 20 ppm for parent masses and 0.1 Da for fragment masses. Carbamidomethylation was set as a permanent modification while deamidation of asparagine and glutamine, N-terminal oxidation of methionine, N-terminal pyroglutamate, C-terminal Lysine loss and proline hydroxylation were set as possible modifications.

### 4.6. EndoS-Mediated Transglycosylation

The transglycosylation reaction was performed by adding 400 µg of plant-made antibody acceptor, 17.5 µg of EndoS D233Q and 40 molar equivalent of complex type (A2G2 as per the Oxford glycan nomenclature, Table S4) N-glycan oxazoline in a 100 mM Tris HCl buffer, pH 7.4. The reaction was incubated at 37 °C for 2 h. Progress of reaction was monitored by LC-ESI-MS analysis of aliquots taken from reaction mixture. Another 40 molar equivalent of oxazoline might be added to reaction mixture for extended incubation if LC-MS monitoring indicated transfer was not complete after 2 h reaction.

### 4.7. Cell Lines and Cell-Based Assay Reagents

A hereditary Spherocytosis cell line Wil2-S (ATCC^®^ CRL-8885) and the Burkitt’s lymphoma cell line Daudi (ATCC^®^ CCL213) were obtained from the American Type Culture Collection (Manassas, VA, USA). Both cell lines were maintained in Gibco^®^ RPMI 1640 medium, supplemented with 10% heat-inactivated fetal bovine serum, penicillin (100 U/mL), streptomycin (100 µg/mL) and non-essential amino acids (Life Technologies, Carlsbad, CA, USA). Cells were maintained with seeding density of 1 × 10^5^ cells/mL and cultured at 37 °C with 5% CO_2_. Cells were subcultured when cell density reached 2 × 10^6^/mL.

### 4.8. Lymphoma B-Cell Lines Characterization

The percentage of CD20 antigen present on cultured Wil2-S and Daudi lymphoma cells was assessed by FACS Calibur flow cytometry (BD Biosciences, San Jose, CA, USA). Daudi and Wil2-S cells at 1 × 10^6^ cells/mL were labeled with FITC Mouse Anti-Human CD20 (BD Biosciences, San Jose, CA, USA). After incubating 100 µL of cells with 5 µL of FITC Mouse Anti-Human CD20 for 30 min at 4 °C, cells were washed and resuspended in stain buffer (Phosphate Buffered Saline (PBS) containing 2% fetal bovine serum) for flow cytometry analysis. The nucleic acid dye 7-Amino-Actinomycin D (7-AAD) (BD Biosciences, San Jose, CA, USA) was used as 0.5 µL/500 µL for the exclusion of nonviable cells in flow cytometry analysis. The relative FITC fluorescence was determined with 488 nm excitation, and emission detected with 530/30 nm using a 530/30 bandpass filter. Percentage of cells with specific CD20-FITC staining was determined with FACS plots using FlowJo FACS analysis software (FlowJo, Ashland, OR, USA).

### 4.9. Antigen Binding Assay

Flow cytometry analysis was performed to determine and compare the binding of _Nb_RTX^A2G2^ and Rituxan^®^ (Genentech, South San Francisco, CA, USA) antibodies to target cells (Wil2-S and Daudi) using a FACS Calibur (BD Biosciences, San Jose, CA, USA). Briefly, 100 µL cells at 1 × 10^6^ cells/mL were incubated with different concentrations of Rituxan^®^ standard and _Nb_RTX^A2G2^ for 30 min at 4 °C. Cells were then washed and incubated with 5 µL of FITC anti-human IgG Fc (BioLegend, San Diego, CA, USA). FITC Mouse IgG2a, k Isotype Ctrl (FC) (BioLegend, San Diego, CA, USA) was used as isotype control. Cells were washed with stain buffer and analyzed by flow cytometry. Nucleic acid dye 7-AAD was used for the quantification of dead cells in each sample as 25 ng/500 µL. Cell Quest data acquisition software (BD Biosciences, San Jose, CA, USA) and Flowjo FACS analysis software (FlowJo, Ashland, OR, USA) were used to derive data plots.

### 4.10. Antibody-Dependent Cell Mediated Cytotoxicity (ADCC) Assay

ADCC was determined using the ADCC Reporter Bioassay Core kit (Promega, Madison, WI, USA) as per manufacturer’s specifications. Wil2-S and Daudi target cells were used along with engineered Jurkat cells stably expressing the FcγRIIIα receptor, V158 (high affinity) and F158 (low affinity) variants. ADCC assays were performed with effector and target cell ratio of 10:1 respectively. In brief, Wil2-S and Daudi target cells were washed with PBS, resuspended in Gibco^®^ RPMI 1640 media supplemented with 2% low IgG serum (Life Technologies, Carlsbad, CA, USA) and seeded at a cell density of 5 × 10^3^ cells/well in white, flat-bottom 96-well assay plates. Experimental wells were set up by combining 25 µL of target cell suspension, 25 µL of effector cell suspension, and 25 µL of the appropriate antibody dilution. Reactions were set up in triplicates and plates were incubated at 37 °C with 5% CO_2_ for 6 h. The Bio-Glo™ Luciferase Assay System was used as detection reagent and luminescence was measured using the Synergy H1 plate reader (Biotek, Winooski, VT, USA). Plate background was determined by calculating the average relative luminescence units (RLU) from wells with media only. GraphPad prism software (GraphPad, La Jolla, CA, USA) was used to plot Relative Luminescence Units (RLU) or Fold of Induction [Fold of Induction = RLU (induced-background)/RLU (no antibody control-background)] vs. Log_10_.

## 5. Patents

Patent US 9,689,016 resulted from the work reported in this manuscript.

## Supplementary Materials

Supplementary materials can be found at http://www.mdpi.com/1422-0067/19/2/421/s1.
