# Supplementary material for: Implementation of Glycan Remodeling to Plant-Made Therapeutic Antibodies
Source: Int J Mol Sci. 2018 Jan 31;19(2):421. doi: 10.3390/ijms19020421 (PMC5855643; doi:10.3390/ijms19020421)
Supplement: Supplementary file 1 [file ijms-19-00421-s001.pdf]

Supplementary Materials: Implementation of Glycan Remodeling to Plant-Made Therapeutic Antibodies

Table S1. Summary of plant-made rituximab heavy chain amino acid modifications detected by LC/MS

	Oxidation	Deamidation	N-terminal pyroglutamate	C-terminal Lysine loss
M469	<0.1%	-	-	-
Q1	-	-	>99.9%	-
N268	-	>99.9%	-	-
N503	-	4.5%	-	-
N532	-	>99.9%	-	-
N578	-	<0.1%	-	-
N601	-	45.6%	-	-
K664	-	-	-	-

Table S2. Summary of plant-made rituximab light chain amino acid modifications detected by LC/MS

	Oxidation	Deamidation	N-terminal pyroglutamate
Q1	-	-	98.8%
N136	-	41.67%	-
N137	-	5.5%	-

Table S3. Calculated masses of unglycosylated plant-made rituximab heavy and light chain based on the observed modifications. [1]plant-made rituximab has a valine residue at position 219 instead of an alanine residue as found on Rituxan®.

	Heavy chain	Light chain
Theoretical mass (unglycosylated)[1]	49,242.5	23,056.7
N-term glutamine cyclization	-17 Da	-17 Da
Deamidation	+2.5 Da	+0.5 Da
Heavy chain C-term Lysine clipping	Not observed	N/A
Heavy chain intra- and intermolecular disulfide bonds (Cysteine n = 11)	-11 Da	/
Light chain intramolecular disulfide bonds (Cysteine n = 5)	/	-5 Da
Expected mass of modified chain	49,217 Da	23,035.2 Da
Calculated mass of unglycosylated antibody $_{Nb}$RTX^{0}	144,504.4 Da	
Calculated mass of deglycosylated antibody $_{Nb}$RTX^{GlcNAc}	144,910.4 Da	

Table S4. Representation of A2G2 glycan structure

Oxford glycan name	Glycan structure
A2G2	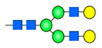

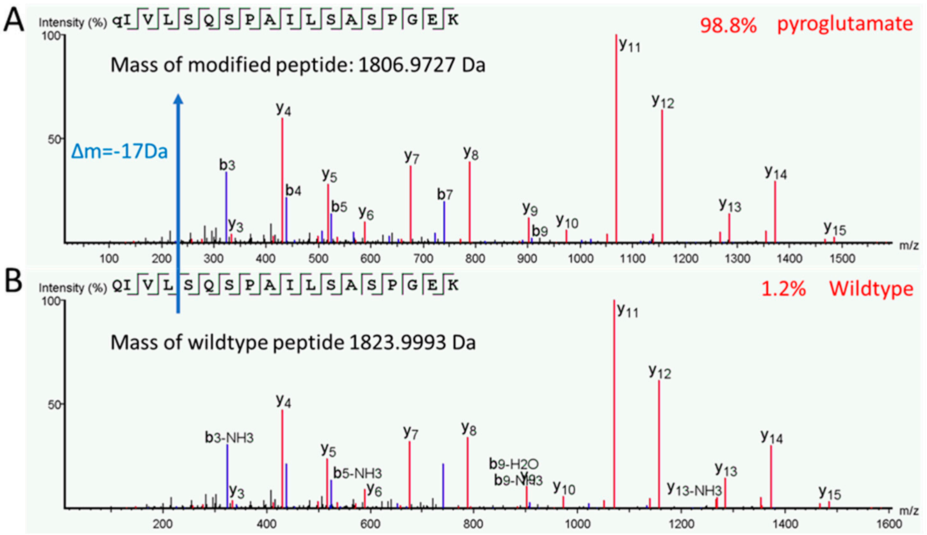

Figure S1. Comparison of the MS/MS spectrum of wildtype and modified peptide [1]QIVLSQSPAILSASPGEK[18]. A) MS/MS spectrum of N-terminal pyroglutamate peptide. B) MS/MS spectrum of wildtype peptide. The mass shift (17Da) of the modified peptide from the wildtype peptide is consistent with an N-terminal pyroglutamate modification. The MS/MS fragmentation pattern of both peptides confirmed the primary sequences. The percentage of N-terminal pyroglutamate peptide was calculated based on MS peak area.

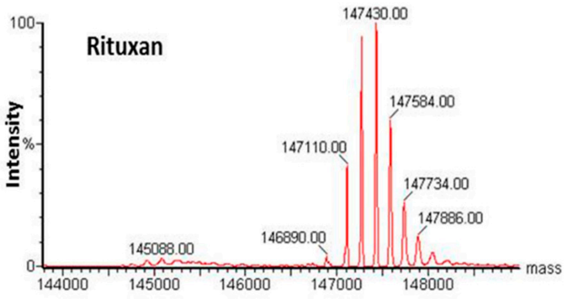

Figure S2. NanoLC-QTOF-MS analysis of innovator protein Rituxan®. Deconvoluted ESI-mass spectrum of Rituxan® analyzed under non-reducing conditions showing a pattern of glycosylated forms (calculated *m/z* of PNGase-F treated Rituxan® 144,190 Da [1])

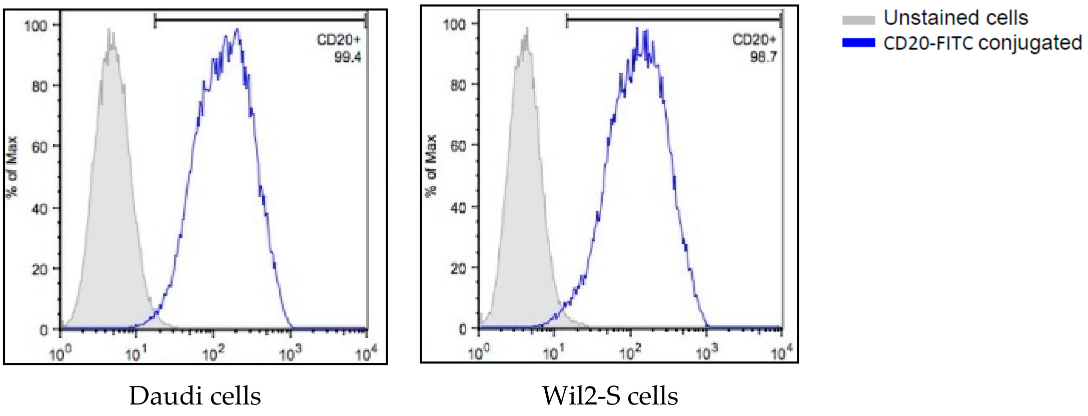

Figure S3: Flow cytometry analysis of CD20 expression on the surface of Wil2-S and Daudi cells. Percentage of CD20 expression is given (upper right corner on each spectrum) and unstained cells are used as control. Fluorescein isothiocyanate (FITC)-conjugated mouse anti-human Fc (blue lines) along with unstained cells (gray solid) are used for the analysis. The X-axis represents the fluorescent signals of FITC whereas the Y-axis presents % of cell count.

Reference

1. Beck, A.; Diemer, H.; Ayoub, D.; Debaene, F.; Wagner-Rousset, E.; Carapito, C.; van Dorsselaer, A.; Sanglier-Cianférani, S. Analytical characterization of biosimilar antibodies and Fc-fusion proteins. *TrAC - Trends Anal. Chem.* **2013**, *48*, 81–95.
